# Augmentation of tibial plateau fractures with an injectable bone substitute: CERAMENT™. Three year follow-up from a prospective study

**DOI:** 10.1186/s12891-015-0574-6

**Published:** 2015-05-13

**Authors:** Riccardo Iundusi, Elena Gasbarra, Michele D’Arienzo, Andrea Piccioli, Umberto Tarantino

**Affiliations:** Department of Orthopedics and Traumatology, University “Tor Vergata”, “Policlinico Tor Vergata” Foundation, Viale Oxford 81, 00133 Rome, Italy; Orthopaedic Department, University of Palermo, Via del Vespro, 90100 Palermo, Italy; Oncologic Center, “Palazzo Baleani”, Azienda Policlinico Umberto I, Corso Vittorio Emanuele II 244, Rome, Italy

**Keywords:** Tibial plateau fracture, Surgical treatment, Bone graft, Ceramic injectable biphasic bone substitute, Clinical and radiographic outcome

## Abstract

**Background:**

Reduction of tibial plateau fractures and maintain a level of well aligned congruent joint is key to a satisfactory clinical outcome and is important for the return to pre-trauma level of activity. Stable internal fixation support early mobility and weight bearing. The augmentation with bone graft substitute is often required to support the fixation to mantain reduction. For these reasons there has been development of novel bone graft substitutes for trauma applications and in particular synthetic materials based on calcium phosphates and/or apatite combined with calcium sulfates. Injectable bone substitutes can optimize the filling of irregular bone defects. The purpose of this study was to assess the potential of a novel injectable bone substitute CERAMENT™|BONE VOID FILLER in supporting the initial reduction and preserving alignment of the joint surface until fracture healing.

**Methods:**

From June 2010 through May 2011 adult patients presenting with acute, closed and unstable tibial plateau fractures which required both grafting and internal fixation, were included in a prospective study with percutaneous or open reduction and internal fixation (ORIF) augmented with an injectable ceramic biphasic bone substitute CERAMENT™|BONE VOID FILLER (BONESUPPORT™, Lund, Sweden) to fill residual voids. Clinical follow up was performed at 1, 3, 9 and 12 months and any subsequent year; including radiographic analysis and Rasmussen system for knee functional grading.

**Results:**

Twenty four patients, balanced male-to-female, with a mean age of 47 years, were included and followed with an average of 44 months (range 41–52 months). Both Schatzker and Müller classifications were used and was type II or 41-B3 in 7 patients, type III or 41-B2 in 12 patients, type IV or 41-C1 in 2 patients and type VI or 41-C3 in 3 patients, respectively. The joint alignement was satisfactory and manteined within a range of 2 mm, with an average of 1.18 mm. The mean Rasmussen knee function score was 26.5, with 14 patients having an excellent result and the remaining 10 with a good result.

**Conclusion:**

It can be concluded that radiological and clinical outcome was satisfactory and obtained in all cases without complications. This injectable novel biphasic hydroxyapatite and calcium sulfate ceramic material is a valuable armamentarium in the treatment of trauma where bone graft is required.

## Background

The goal of tibial plateau fracture management is a stable, well aligned, congruent joint, with a painless range of motion and function [1]. In the analysis of the treatment of 60 patients with tibial plateau fractures Blokker *et al.* [2] concluded that the single most important factor in predicting outcome was the fracture reduction. Open reduction and internal fixation (ORIF) with standard plates and screws or screws alone does not always mantain the reduction [3, 4]. After restoration of a congruent joint surface bone grafting and buttress plating are often needed to allow early mobility and weight bearing [1].

Bone grafting is thus often needed following reduction of the tibial fracture to fill residual metaphyseal voids and gaps. Autograft is considered ideal for grafting procedures, providing osteoinductive growth factors, osteogenic cells being an osteoconductive scaffold [5] but is associated with morbidity at the donor site and is limited in supply. Allograft has been employed as a good alternative to autograft in the treatment of depressed tibial fractures [6] but the logistics of supply and the concern of potential disease transmission remains [7]. Synthetic bone graft substitutes have been gaining popularity as viable alternatives for void and defect filling eliminating the concerns with autograft and allograft. These synthetic bone substitutes have invariably been based on calcium phosphate and/or calcium sulfate materials which are osteoconductive and facilitate bone remodeling.

We present here the three year radiological and functional results of a prospective series of tibial plateau fractures treated with closed or open reduction, internal fixation and augmentation with an injectable novel hydroxyapatite and calcium sulfate bi-phasic ceramic bone substitute, CERAMENT™.

## Methods

During 12 months from June 2010 through May 2011 adult patients between 18 and 70 years, presenting with acute, closed and unstable proximal tibial fractures which required both grafting and internal fixation, were included in a prospective study with percutaneous or open reduction and internal fixation (ORIF) augmented with an injectable ceramic biphasic bone substitute CERAMENT™|BONE VOID FILLER (BONESUPPORT™, Lund, Sweden) to fill residual gaps. Excluded from the study were patients with metabolic bone disease, type 1 diabetes or uncontrolled type 2 diabetes, malignancy or on treatment with systemic steroids or immunosuppressive therapy, infection at the operative site, concurrent treatment with other bone substitutes including autograft, peripheral vascular disease, alcoholism, substance abuse, correlated peripheral nerve damage, pregnancy or breast feeding or fertile women not on routine contraceptive control, a history of anaphylactic reaction to iodine-based radiocontrast agents, known bleeding disorders, hyperthyreosis and thyroid adenoma. Tobacco use was not an exclusion criteria. This investigation was performed in accordance with the ethical standards of the Declaration of Helsinky and all the patients gave informed consent prior to being included in the study but local ethics committee authorization was not required because the material was regarded to the standard of care.

The flowable and *in situ* curing bio-ceramic bone substitute was injected under fluoroscopy with a minimally invasive technique from the contralateral side of the fractured tibia via the bony window used for reduction (all patients of Schatzker type II or Müller 41-B3 and 8 patients out of type III or 41-B2) and internal fixation was applied. The material consists of highly osteoconductive hydroxyapatite particles embedded in a calcium sulphate (CaS) paste, which is prepared 3 minutes before application by mixing with the water-soluble radiocontrast agent, iohexol, in a closed system. Once implanted, CERAMENT™ has a compressive strength of at least 5 MPa, which is comparable to that of healthy trabecular cancellous bone. Due to its similarity to endogenous bone, the CERAMENT™ implant triggers an endogenous precipitation of hydroxyapatite on its surface which prevents passive resorption. The sulphate dehydrate part of the implant is gradually resorbed during 7–8 weeks and being replaced by ingrowing bone that remodels to form trabecular bone, supplied by the remaing hydroxyapatite nanoparticles thus incorporated in to the newly formed trabecular bone [8]. Progressive load bearing was allowed after 2 weeks for Schatzker II and III or Müller AO type 41-B fractures and after 4 weeks for type IV and VI or type 41-C fractures.

Clinical follow up was performed at 1, 3, 9 and 12 months and any subsequent year; including radiographic analysis and knee functional grading. X-ray review allowed the assessment of osteosynthesis stability, preserved alignment of the joint surface following reduction, bone substitute resorption and fracture healing. The Rasmussen system [9] was employed for knee functional grading. A CT scan was taken preoperatively to examine the fracture, for surgery planning and for fracture classifications according to Schatzker [10] and Müller [11].

## Results

Ninety-three consecutive patients with a tibial plateau fracture presented at the Emergency Department of the University Hospital “Tor Vergata” over the 12 month recruitment period. A total of 24 patients, 12 males and 12 females, met the inclusion criteria and were included and treated by three of the authors: the patients ranged from 32 to 64 years, mean 47 years. Schatzker classifications were type II (7 patients), type III (12 patients), type IV (2 patients) and type VI (3 patients); Müller classifications were as follows: 41-B2 (12 patients), 41-B3 (7 patients), 41-C1 (2 patients) and 41-C3 (3 patients). Fifteen patients (all type II or 41-B3 and 8 patients out of type III or 41-B2) were operated with two percutaneous cannulated screws which were inserted through the lateral side after that a distal medial window to reduce the fracture first and to introduce the bone substitute later, was performed; the remaining 9 patients (4 patients out of type III or 41-B2, all type IV or 41-C1 and all type VI or 41-C3) needed an angled stable sliding plate with screws and the bone substitute was inserted directly by the lateral approach.

The interim follow-up period was an average of 44 months (41–52 months) as presented in Table 1. Radiographic analysis demonstrated that a loss of fracture reduction was maintained within the satisfactory range of 2 mm, with an average of 1.18 mm as presented in Table 2. The CERAMENT™ resorption was complete in all cases after an average of 5 months (3–8 months).

**Table 1:** Demographics, Schatzker and Müller classifications and type of fixation

Age (years)	Mean 47 (range 32–64)
Male gender	50 %
Schatzker classification	N = 7 II; N = 12 III; N = 2 IV; N = 3 VI
Müller classification	N = 12 41-B2; N = 7 41-B3; N = 2 41-C1; N = 3 41-C3
Screw fixation	15 pts
Plate fixation	9 pts

pts, patients

**Table 2:** Post-operative radiographic assessment

Reduction: overall mean step-off	Patients	Mean step-off	Schatzker classification	Müller classification
1.18 mm	16	≤1.2 mm	N = 4 II; N = 12 III	N = 12 41-B2; N = 4 41-B3
	5	1.2 ≤ 1.5 mm	N = 3 II; N = 2 VI	N = 3 41-B3; N = 2 41-C3
	2	1.6 mm	N = 2 IV	N = 2 41-C1
	1	≈1.7 mm	N = 1 VI	N = 1 41-C3

**Fig. 1:**
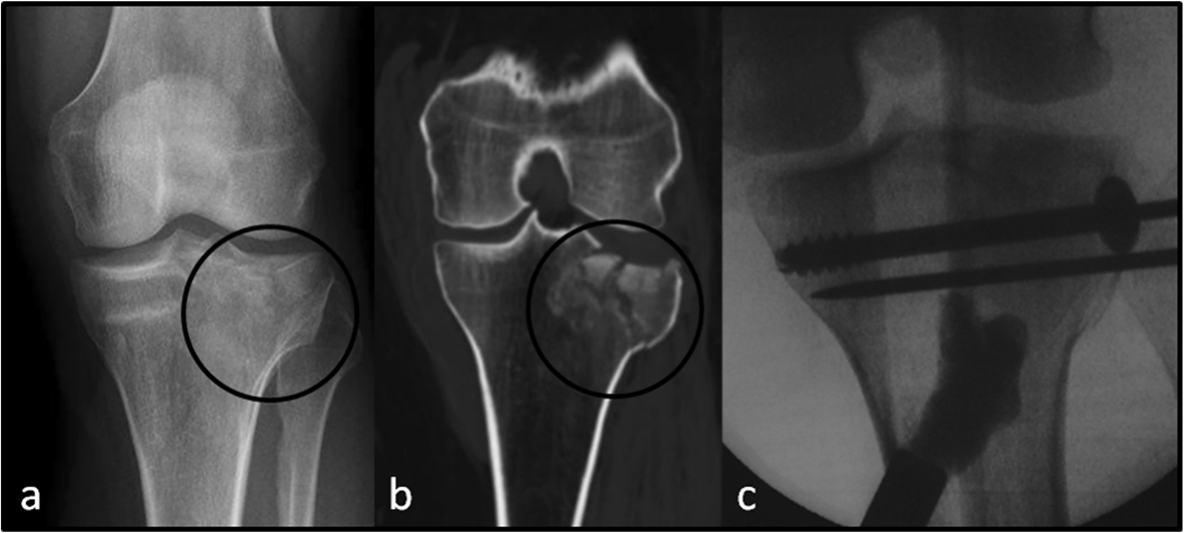
A 35 year old female with a Schatzker II, Müller AO grade 41-B3 fracture. (**a**) Pre-operative X-rays; (**b**) Pre-operative CT-scan; (**c**) Intra-operatively CERAMENT™ was injected under fluoroscopy following closed reduction and before fixation with two cannulated cancellous bone screws

**Fig. 2:**
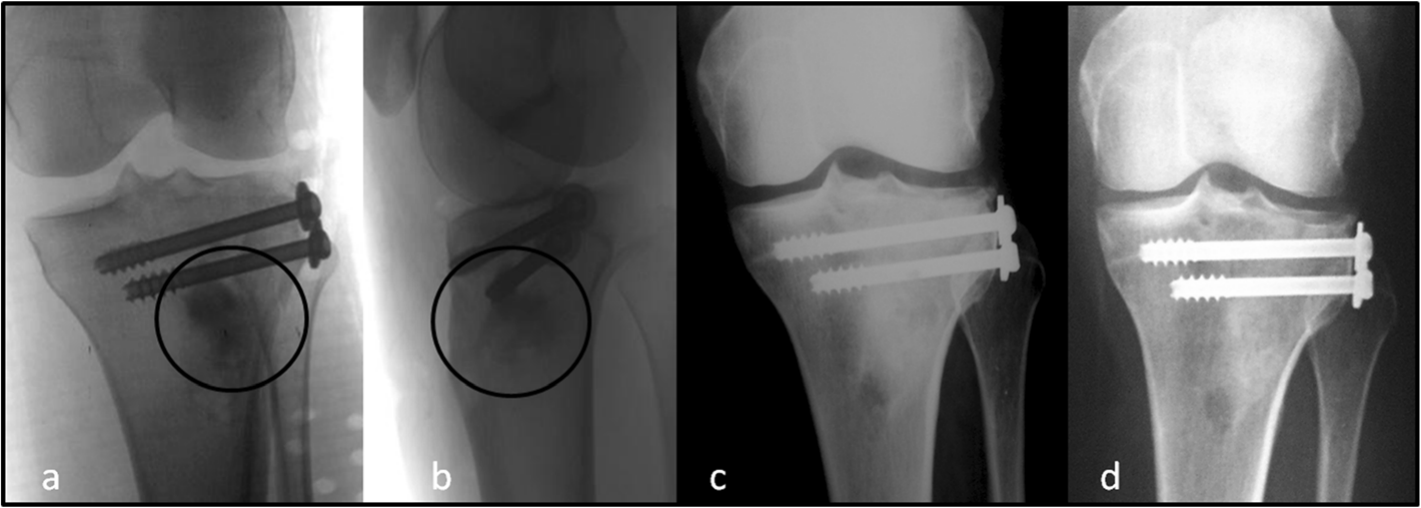
X-rays follow-up at different steps. (**a, b**) The positioning of the bioceramic may been seen on the immediate post-operative frontal and sagittal x-rays; (**c**) On the same views at 1 month post-op there is no further evidence of radiocontrast of the bone cement indicating the washout of iohexol; (**d**) The remodeling process is now progressing. New bone appears well consolidated at 3 months

**Fig. 3:**
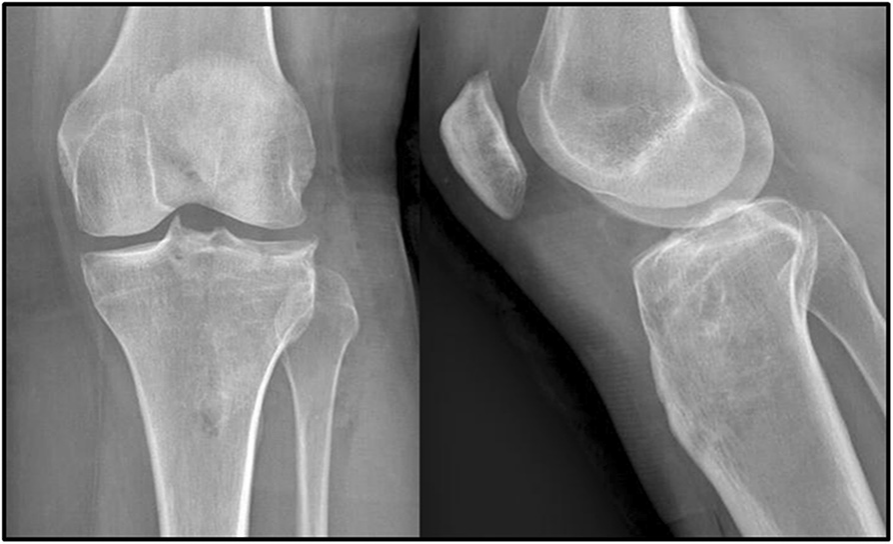
Excellent bone regeneration with the removal of the hardware after 9 months since osteosynthesis

The mean Rasmussen knee function score was 26.5, with 14 patients exhibiting an excellent result and the remaining 10 with a good rating. A case of a 35 year old female with a Schatzker II, Müller AO grade 41-B3 fracture, is presented in Figs. 1, 2 and 3.

## Discussion

Following reduction of tibial fractures bone graft is often needed to fill residual voids and gaps. Autograft and allograft have been used for this purpose but there exists disadvantages that cannot be overlooked, for instance donor site pain and morbidity, blood loss, infection, slow rate of incorporation and secondary fractures. For these reasons there has been an emergence of bone graft substitutes for trauma applications and in particular synthetic materials based on calcium phosphates and/or apatite calcium sulfates. Synthetic bone substitutes provide a ready and plentiful supply of material and injectable versions optimize the filling of irregular bone defects. Ozturkmen *et al.* [12] in the treatment of 28 patients with ORIF and a calcium phosphate based bone cement found that the time to full weight bearing was reduced to less than 6.5 weeks. In a clinical series of 43 patients with trauma fractures McAndrew *et al.* [13] demonstrated the efficacy of a calcium phosphate based bone substitute at a 6 to 12 month follow-up, with 90 % in a fracture group and 85 % in a non-union group demonstrating healing. A meta-analysis of randomized clinical trials conducted by Bajammal *et al.* [14] in a series of metaphyseal fractures involving tibial plateau, femoral neck, intertrochanteric femoral and calcaneal indications, concluded that loss of fracture reduction was less with a calcium phosphate based bone filler in comparison with autogenous bone graft. Bucholz *et al.* [15] found no significant differences in radiographic and clinical assessments between a calcium phosphate based bone cement and autograft for residual void and gap in a series of 40 patients with displaced tibial fracture.

In cadaveric pre-clinical investigations [16–18] calcium phosphate based cements used to fill proximal tibial defects following trauma were found to be stronger than autologous graft filling with greater stiffness, higher fatigue strength and a greater ultimate load supporting capacity. This is supported clinically by Simpson and Keating [19] at 1 year in 26 patients in an equally balanced study with the use of autograft and a calcium phosphate based bone substitute for the treatment of proximal tibial fractures. The mean residual plateau depression was 4 mm in the autograft group (coupled with buttress plating) and 0.7 mm in the calcium phosphate based group (with minimal internal fixation) at 1 year follow-up. They also were able to use less hardware with the calcium phosphate group and had a corresponding 40 % reduction in operative time on average (from a mean of 101 min to 55 min). Larsson and Hammick [20] in their analysis of randomized clinical trauma studies support the benefits of injectable calcium phosphate based bone substitutes as suitable alternatives to autograft, with the flexibility to be applied around the hardware once in position and to augment weaker bone around the screws as well as replace autograft in defects and voids.

It can be concluded from our preliminary review that radiological and clinical outcome was satisfactorily obtained in all cases without complications. The enhanced stability with the application of CERAMENT™ supported and maintained fracture reduction with a mean setting of 1.18 mm at an average of 9 months in the current clinical series, well within the satisfactory range of 2 mm and in line with the findings of Simpson and Keating [19]. The Rasmussen knee functional score had all patients in the good and excellent categories demonstrating results at least as good as those for Ozturkmen *et al.* [12] who employed calcium phosphate cement augmentation in their treatment of depressed tibial fractures and returned 78 % of patients back to their pre- operative level of activity.

CERAMENT™ is designed to remodel in tune with the natural bone remodelling process. Due to the microporosity through the release of the CaS component, an immediate flow of tissue fluids with nutrients and growth factors are allowed to penetrate the implant, which promotes osteoclasts and macrophages to enter the material and create macropores resulting in a widespread ingrowth of early bone. The end result is full transformation and remodeling into mature bone after 9–12 months [21, 22]. This interim study at an average of 44 months follow-up of the bone remodeling process shows that new normal bone is formed and maintained which is important for clinical performance in the longer term.

This investigation has some limitations. The study population is relatively small and doesn’t effectively represent all types of tibial plateau fractures. Three out of the authors performed the surgical procedures. Next, a control group using other bone substitutes or grafting was not enrolled. Lastly, patients were followed for a mid-period, so further studies will be required to verify the long-term clinical and radiological outcomes.

## Conclusion

CERAMENT™|BONE VOID FILLER has demonstrated that it is a viable replacement to bone grafting in filling voids and gaps following fracture of the tibial plateau. It provides a material, being injectable which optimizes the filling of irregular bone defects. This study has demonstrated that CERAMENT™ has supported the reduction and preservation of alignment of the joint surface until fracture healing. This makes the novel biphasic bio-ceramic material an attractive solution in the treatment of trauma situations where bone graft is required.
